# The Vibration of a Linear Carbon Chain in Carbon Nanotubes

**DOI:** 10.3390/ma10050478

**Published:** 2017-04-29

**Authors:** Dongqing Ding, Yang Zhao, Shuhong Dong, Peishi Yu, Liqiang Wang, Junhua Zhao

**Affiliations:** Jiangsu Key Laboratory of Advanced Food Manufacturing Equipment and Technology, Jiangnan University, Wuxi 214122, China; ddqjnu@163.com (D.D.); zhaoyang479218533@126.com (Y.Z.); dsh@jiangnan.edu.cn (S.D.); ypsnuaa@163.com (P.Y.)

**Keywords:** carbon chain, vibration, carbon nanotube, van der Waals interactions

## Abstract

An explicit solution for the vibration of a carbon chain inside carbon nanotubes (CNTs) was obtained using continuum modeling of the van der Waals (vdW) interactions between them. The effect of the initial tensile force and the amplitude of the carbon chain as well as the radii of the CNTs on the vibration frequency were analyzed in detail, respectively. Our analytical results show that the vibration frequency of the carbon chain in a (5,5) CNT could be around two orders of magnitude higher than that of an independent carbon chain without initial tensile force. For a given CNT radius, the vibration frequency nonlinearly increases with increasing amplitude and initial tensile force. The obtained analytical cohesive energy and vibration frequency are reasonable by comparison of present molecular dynamics (MD) simulations. These findings will be a great help towards understanding the vibration property of a nanowire in nanotubes, and designing nanoelectromechanical devices.

## 1. Introduction

Carbyne is an allotrope of carbon composed of *sp*-hybridized carbon atoms [1,2,3]. The longest polyynes consisting of 44 contiguous acetylenic carbons inside thin double-walled (DW) CNTs with alternation single and triple bonds were firstly synthesized by Chalifous and Tykwinski [4]. Afterwards, Shi et al. further reported the considerably long acethylenic linear carbon chains (more than 10,000 carbons) in thin DWCNTs under very high temperature and high vacuum conditions by using a novel experimental method [5]. These findings of linear carbon chain inside CNTs have attracted considerable research interests because of their potential applications in nanoeletronic devices. To design and set up the reliable nanoelectronic devices, the mechanical property of a carbon chain by considering the adhesive effect between a carbon chain and a CNT is a critical issue and should be elucidated in detail. Liu et al. reported that Carbyne’s strength, the elastic modulus, and the stiffness are greater than those of any known material, including diamond, CNTs, and graphene using first-principles calculations [6]. Hu et al. studied the enhanced critical pressure for buckling CNTs in view of an inserted linear carbon chain [7], and the effect of the inserted carbon chain on the vibration of a CNT was then further investigated [8]. 

However, the cohesive energy between a carbon chain and a CNT is still not clear due to the van der Waals (vdW) interactions, which could strongly affect the vibration property of the carbon chain for a thin CNT.

In this study, closed-form expressions for the vibration of a carbon chain inside CNTs were obtained using continuum modeling of the vdW interactions between them. The effect of the initial tensile force, the amplitude of the carbon chain, and the radii of the CNTs on the vibration frequency were studied in detail. Checking against present MD simulations shows that the continuum solutions of the cohesive energy have high accuracy, while the comparison of the vibration frequency is reasonable.

## 2. Continuum Modeling and Molecular Dynamics Simulation

The energy of the vdW interactions between two atoms is given by [9]

(1) $$V\left(r\right)=4\in \left[{\left(\frac{\sigma }{r}\right)}^{12}-{\left(\frac{\sigma }{r}\right)}^{6}\right]$$

where *r* is the distance between the interacting atoms, ∈ is the depth of the potential, and σ is a parameter that is determined by the equilibrium distance. In this paper, ∈ = 2.8437 mev and *σ* = 3.4 Å are adopted from the literature [10,11].

**Figure 1:**
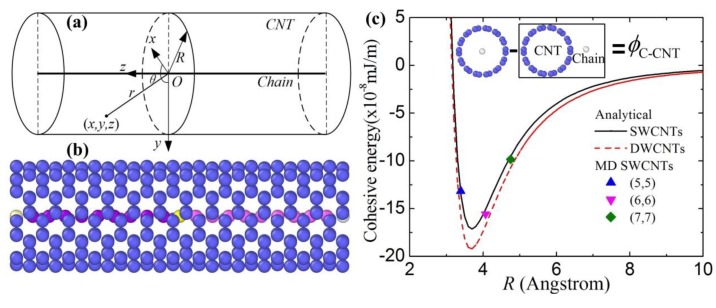
A coordinate system and a schematic diagram as well as cohesive energy of a carbon chain in an SWCNT. (**a**) The coordinate system and the schematic diagram. (**b**) The atomic structures of a carbon chain in a (5,5) CNT. (**c**) The cohesive energy of a carbon chain in different SWCNTs and DWCNTs using continuum modeling and MD simulations.

We homogenize carbon atoms on the CNT and represent them by an area density *ρ*_g_, which is related to the equilibrium bond length of graphene prior to deformation. From the unit cells and bond lengths, the area density *ρ* can be expressed as $\rho_{g}=4/\left[3\sqrt{3}b^{2}\right]$, where *b* = 1.42 Å is the bond length of the graphene sheets [12,13]. The line density of the carbon chain is *ρ*_c_ (the average number of atoms per unit length). The Cartesian coordinates (*x*, *y*, *z*) is shown in Figure 1a, where *z* is along the central axis of the nanowire and *y* is normal to the CNT. Without loss of generality, the cohesive energy per unit line of the interface between the carbon chain and the CNT can be given as

(2) $$\phi_{c-\mathrm{CNT}}=\rho_{c}\rho_{g}\int_{0}^{2\pi }Rd\theta \int_{-\infty }^{+\infty }V(r)dz$$

where $r^{2}={\left(R\cos\theta \right)}^{2}+{\left(R\sin\theta \right)}^{2}+z^{2}$.

Equation (2) can be expressed as

(3) $$\phi_{c-\mathrm{CNT}}=8\pi \varepsilon \rho_{c}\rho_{g}\sigma^{6}\left[\sigma^{6}\frac{63\pi }{256}\frac{1}{R^{10}}-\frac{3\pi }{8}\frac{1}{R^{4}}\right].$$

To validate the results of the continuum modeling, the cohesive energy and vibration frequency of a carbon chain inside CNTs are obtained by MD simulations (see Figure 1b). In present MD simulations, the total energy is minimized by the conjugate-gradient algorithm. A carbon chain and a CNT are considered as two rigid bodies in order to obtain the cohesive energy. All the MD simulations are performed using LAMMPS [14] with the AIREBO potential [15] (CNTs) and harmonic potential [16] (carbon chain). The total energy of a chain inside a CNT minus the total energy of a chain outside a CNT derives the cohesive energy in Figure 1c. The cohesive energy of present MD simulations agrees well with that of Equation (3), in which the difference is less than 3%. The carbon chain is subjected to the repulsive force from the CNT when the CNT radius is lower than 1.086 *σ* from $\partial \phi_{c-\mathrm{CNT}}/\partial R=0$ (see Figure 1c), while the attractive force dominates the carbon chain from the CNT when the CNT radius is higher than 1.086 *σ*. Moreover, the cohesive energy of a carbon chain inside a DWCNT is also obtained in Figure 1c. The red dash line represents the radius distribution of the inner CNT of the DWCNT. The difference of the cohesive energy inside an SWCNT is very small (<10%) by comparison to that inside a DWCNT when the radius of the SWCNT is identical with that of the inner CNT of the DWCNT.

**Figure 2:**
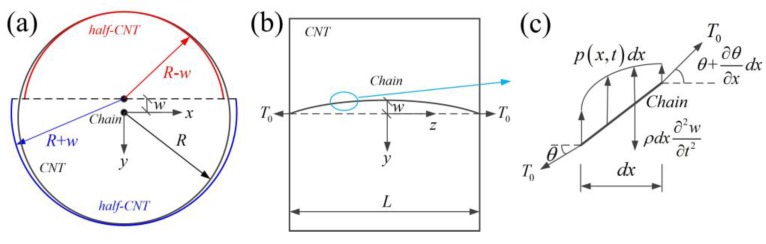
(**a**) The moving position of a point on a carbon chain in view of the carbon chain vibration in an SWCNT; (**b**) The side view of the carbon chain under initial tension in (a); (**c**) The vdW force distribution on the carbon chain under initial tension.

To obtain the vibration frequency of the carbon chain, the two ends of the carbon chain are always simply supported and the middle atom moves to 0.001 Å along the out-of-plane direction at each time step (the time step is 0.1 fs) based on the displacement-control method (the structure is optimized for each displacement increment). When the middle part moves to a given amplitude (see Figure 2a,b), the vibration is generated by allowing the middle part to evolve freely at a later time (see Figure 2c). Note that the CNT is always fixed. The out-of-plane displacement of the carbon chain in the vibration process could strongly affect the distribution of the vdW force, which dominates the vibration frequency.

From Equation (3), the vdW force of the carbon chain per unit length after vibration can be obtained:

(4) $$F_{vdw}=\int_{0}^{\pi }\frac{1}{2\pi R}\frac{\partial \phi_{c-\mathrm{CNT}}}{\partial R}\sin\theta Rd\theta =8\varepsilon \rho_{c}\rho_{g}\sigma^{6}\left[-10\sigma^{6}\frac{63\pi }{256}\frac{1}{R^{11}}+4\frac{3\pi }{8}\frac{1}{R^{5}}\right].$$

To study the chain vibration, the carbon chain can be assumed as a string. The function of the string vibration can be expressed as

(5) $$\rho \frac{\partial^{2}w}{\partial t^{2}}=T_{0}\frac{\partial \theta }{\partial x}+p\left(x,t\right)$$

where ρ is the mass of the carbon chain per unit length, $T_{0}$ is the initial tensile force, p(x,t) is the stress of the carbon chain per unit length, and *w* is the deflection of the carbon chain.

Assuming $a=\sqrt{\frac{T_{0}}{\rho }}$, substituting $\theta =\frac{\partial w}{\partial x}$ into Equation (5) gives

(6) $$\frac{\partial^{2}w}{\partial t^{2}}=a^{2}\frac{\partial^{2}w}{\partial x^{2}}+\frac{1}{\rho }p\left(x,t\right)$$

where p(x,t)=Cw, C=DK, $D=D_{1}+D_{2}$ and

$$D_{1}=\sum_{n}\frac{\partial F_{vdw}}{\partial R}{|}_{R=R_{n}-A}=8\varepsilon \rho_{c}\rho_{g}\sigma^{6}\left[110\sigma^{6}\frac{63\pi }{256}\frac{1}{{\left(R_{n}-A\right)}^{12}}-20\frac{3\pi }{8}\frac{1}{{\left(R_{n}-A\right)}^{6}}\right]$$

$$D_{2}=\sum_{n}\frac{\partial F_{vdw}}{\partial R}{|}_{R=R_{n}+A}=8\varepsilon \rho_{c}\rho_{g}\sigma^{6}\left[110\sigma^{6}\frac{63\pi }{256}\frac{1}{{\left(R_{n}+A\right)}^{12}}-20\frac{3\pi }{8}\frac{1}{{\left(R_{n}+A\right)}^{6}}\right]$$

$$K=\frac{\int_{0}^{L}wdx}{AL}=\frac{\int_{0}^{L}A\sin\left(\frac{\pi x}{L}\right)dx}{AL}=\frac{2}{\pi }$$

where *D*_1_ and *D*_2_ represent the modified coefficient of vdW interactions between the carbon chain to upper half-CNT and lower half-CNT (see Figure 2a). In the vibration process, the vdW interactions are asymmetrical between the carbon chain to CNT, so we approximately use two half-CNTs with the two radii of *R* − *w* and *R* + *w* to calculate the asymmetric interactions (see Figure 2a). A is the maximum deflection of the string. *K* represents the modified coefficient of vdW interactions due to the different deflection of each position on the string in the vibration process, and L is the length of the carbon chain (in the context of this paper, we choose the length *L* as 30 Å for the carbon chain).

If we suppose that the two ends of the above nonlocal beam are simply supported, then the deflection of the beam can be expressed as

(7) $$w=A\sin\left(\frac{\pi x}{L}\right)e^{i\omega t}.$$

The vibration frequency ω can be obtained by substituting Equation (7) into Equation (6):

(8) $$\omega =\sqrt{a^{2}{\left(\frac{\pi }{L}\right)}^{2}-\frac{1}{\rho }C}.$$

**Figure 3:**
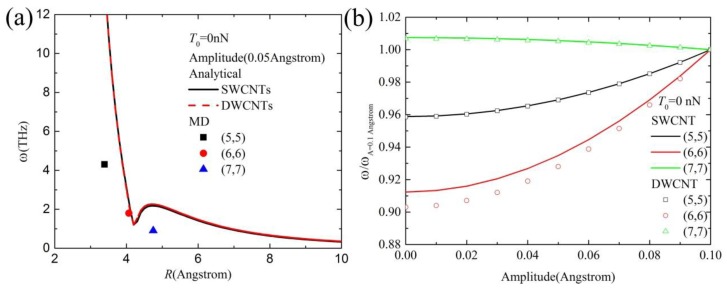
(**a**) The vibration frequency distribution of a carbon chain in different SWCNTs and DWCNTs by continuum modeling and MD simulations; (**b**) The amplitude effect on the normalized vibration frequency distribution.

Figure 3a shows the vibration frequency distribution of a carbon chain (inside different SWCNTs and DWCNTs) with CNT radii by continuum modeling and MD simulations without initial tensile force (*T*_0_ = 0). The difference inside SWCNTs and DWCNTs for a given same SWCNT radius (or an inner CNT radius) and amplitude (0.05 Å) is considerably small, which can be neglected in Figure 3a. The obtained vibration frequency from Equation (8) is reasonable by comparison with the vibration frequency of our MD simulations. When the CNT radius *R* is higher than 10 Å or *R* = *R*_0_ (*R*_0_ can be obtained by ∂D/∂R=0 of Equation (6), where *R*_0_ is also the function of amplitude), the vdW force between the carbon chain and the CNT is too small and can be neglected. That is to say, the vibration of the carbon chain is close to a free vibration. Figure 3b shows the amplitude effect of the carbon chain inside different SWCNTs and DWCNTs on its normalized vibration frequency distribution for *T*_0 _= 0, where *ωA* = 0.1 angstrom represents the vibration frequency of the chain inside different CNTs when the amplitude is given as 0.1 Å. The normalized vibration frequency nonlinearly increases with increasing amplitude. The change rate of the vibration frequency in (5,5) CNT with increasing amplitude (0.005~0.1 Å) is almost the same as that in (6,6) CNT, while the change rate in (7,7) CNT is higher than that in (5,5) CNT. The probable reason is that the absolute value of the repulsive force of the carbon chain in (5,5) CNT is almost identical with that of the attractive force in (6,6) CNT in Figure 1c. 

**Figure 4:**
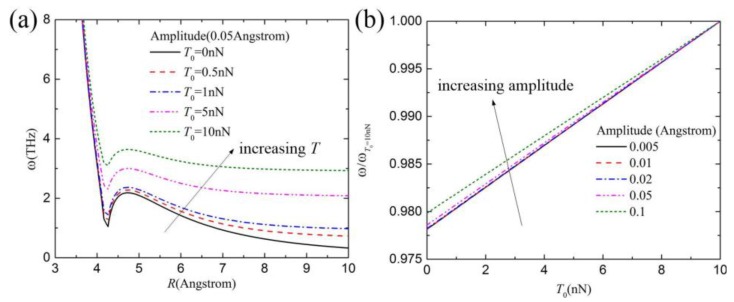
(**a**) The radii effect of SWCNTs on the vibration frequency distribution of a carbon chain in different SWCNTs; (**b**) The effect of the initial tensile force on the normalized vibration frequency distribution of a carbon chain in (5,5) SWCNT.

Figure 4a shows the vibration frequency distribution of the carbon chain with SWCNT radius for a given amplitude (0.05 Å), in which the carbon chain is subjected to different initial tensile forces. The vibration frequency nonlinearly increases with increasing initial tensile force. Figure 4b shows the effect of the initial tensile force on the normalized vibration frequency distribution of a carbon chain in (5,5) SWCNT. For higher amplitude, the normalized vibration frequency is closer to a linear increase with increasing initial tensile force. The possible reason is that the repulsive force dominates the vibration frequency when the amplitude is very high, while both the repulsive force and the attractive force dominate the frequency for smaller amplitude.

## 3. Discussion

The vdW force between a carbon chain and a CNT plays a key role in the vibration frequency of the chain. A fundamental understanding of mechanical vibration of the carbon chain inside a CNT is crucial for their potential applications in designing nanoelectromechanical systems and electronic devices. 

In this paper, the vibration frequency of a carbon chain inside a CNT is obtained using continuum modeling, in which the carbon chain is taken as a string. It should be stressed that the effect of the vdW force between the carbon chain and the CNT on CNT deformation is neglected here. In other words, the CNT is taken as a rigid body. 

**Figure 5:**
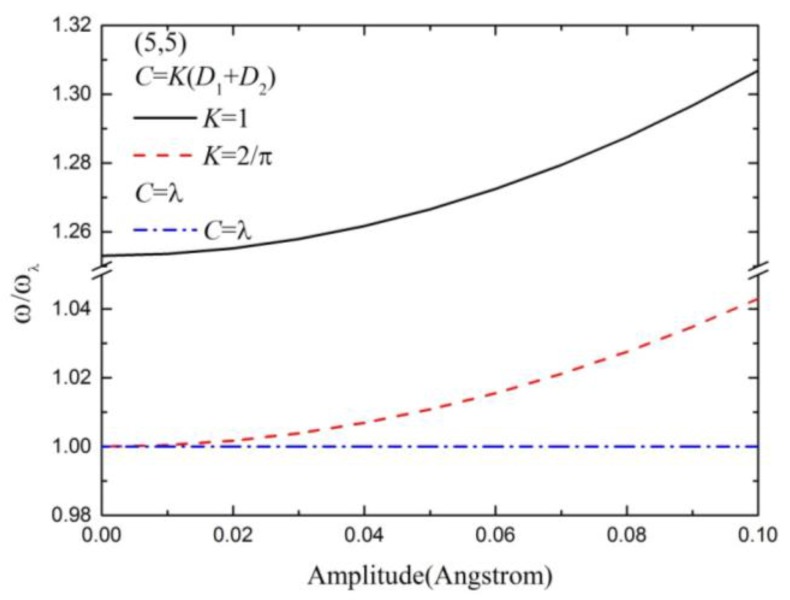
The normalized vibration frequency distribution with different amplitudes by using three modified coefficients of vdW interactions for a carbon chain inside (5,5) CNT.

Furthermore, Equation (6) cannot be solved when *D*_1_ and *D*_2_ is taken as a function of *w*, so *D*_1_ and *D*_2_ are assumed as the function of *A* which is the maximum value of *w*. In this method, the vibration frequency should be the maximum value in Equation (6), and the modified coefficient of vdW interactions then becomes *C* = *D* = *D*_1_ + *D*_2_ (see Equation (6)). Due to the different deflection of each point on the string in the vibration process, we replace the amplitude *w* by average amplitude $\int_{0}^{L}wdx/L$, and represent the modified coefficient of vdW $K=\int_{0}^{L}wdx/AL$ interactions. Thus, the modified coefficient of vdW interactions becomes *C* = (*D*_1_ + *D*_2_)*K*. When a carbon chain is located in the center of CNT, *A* = 0 results in *D*_1_ = *D*_2_ in Equation (6). Here, we define *C* = *λ*= 2*D*_1_. Figure 5 shows the normalized vibration frequency distribution of the carbon chain with different amplitudes for a given (5,5) CNT, in which the three modified coefficients of vdW interactions are used. All normalized vibration frequencies nonlinearly increase with increasing amplitude. For the case of *C* = *λ*, the vibration frequency is the minimum vibration frequency. For the case of *K* = 1, the vibration frequency is the maximum vibration frequency. The realistic values of the vibration frequency should be between the two values. In this work, the modified coefficient *K* = 2/*π* should be closer to the realistic values. 

In addition, it should be stressed that the bending stiffness of the carbon chain has some effect on the vibration, which is also ignored in this paper. In this work, the carbon chain is taken as a string. Equation (7) is obtained when the two ends of the string is simply supported. Since the bending stiffness of the string is neglected, the carbon–carbon interaction within the carbon chain cannot be exactly described in this boundary condition. We use the string model to study the vibration here since the cross-sectional area of the carbon chain is not easy to determine. Of course, the beam model can also be used to describe the effect of the bending stiffness on the vibration, but the vibration equation based on the modified vdW interactions (*D*_1_ and *D*_2_ in Equation (6)) cannot be solved. The issue should be more useful in practical applications and will be further studied in the next work.

## 4. Conclusions

In this study, explicit equations for the vibration of a carbon chain inside CNTs were derived using continuum modeling of the vdW interactions between them. The effect of the initial tensile force, the amplitude of the carbon chain, and the radii of the CNTs on the vibration frequency were obtained. The conclusion can be summarized as follows:

(1) The vibration frequency of the carbon chain in a (5,5) CNT could be around two orders of magnitude higher than that of an independent carbon chain without initial tensile force.
(2) For a given CNT radius, the vibration frequency nonlinearly increases with increasing amplitude and initial tensile force.
(3) By comparison of our MD simulations, the current analytical solution of the vibration frequency is reasonable, which should be of great help towards understanding the vibration property of a carbon chain in CNTs.
